# Fibro-adipogenic progenitors in skeletal muscle homeostasis, regeneration and diseases

**DOI:** 10.1098/rsob.210110

**Published:** 2021-12-08

**Authors:** Thomas Molina, Paul Fabre, Nicolas A. Dumont

**Affiliations:** ^1^ CHU Sainte-Justine Research Center, Montreal, Quebec, Canada; ^2^ Department of Pharmacology and Physiology, Faculty of Medicine, Université de Montréal, Montreal, Quebec, Canada; ^3^ School of Rehabilitation, Faculty of Medicine, Université de Montréal, Montreal, Quebec, Canada

**Keywords:** fibro-adipogenic progenitors, mesenchymal stromal cells, muscle stem cells, myogenesis, fibrosis, muscular disorders

## Abstract

Skeletal muscle possesses a remarkable regenerative capacity that relies on the activity of muscle stem cells, also known as satellite cells. The presence of non-myogenic cells also plays a key role in the coordination of skeletal muscle regeneration. Particularly, fibro-adipogenic progenitors (FAPs) emerged as master regulators of muscle stem cell function and skeletal muscle regeneration. This population of muscle resident mesenchymal stromal cells has been initially characterized based on its bi-potent ability to differentiate into fibroblasts or adipocytes. New technologies such as single-cell RNAseq revealed the cellular heterogeneity of FAPs and their complex regulatory network during muscle regeneration. In acute injury, FAPs rapidly enter the cell cycle and secrete trophic factors that support the myogenic activity of muscle stem cells. Conversely, deregulation of FAP cell activity is associated with the accumulation of fibrofatty tissue in pathological conditions such as muscular dystrophies and ageing. Considering their central role in skeletal muscle pathophysiology, the regulatory mechanisms of FAPs and their cellular and molecular crosstalk with muscle stem cells are highly investigated in the field. In this review, we summarize the current knowledge on FAP cell characteristics, heterogeneity and the cellular crosstalk during skeletal muscle homeostasis and regeneration. We further describe their role in muscular disorders, as well as different therapeutic strategies targeting these cells to restore muscle regeneration.

## Graphical abstract

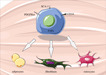

## 1. Introduction

With more than 600 skeletal muscles and accounting for 35–45% of the body mass, striated skeletal muscle is the most abundant tissue in the human body [1]. Skeletal muscles play a role in vital functions, such as locomotion, breathing, thermoregulation, energy metabolism and endocrine signalling. Considering their role in movement and exercise, skeletal muscles are subjected to various physical stresses and traumas. To face these challenges, skeletal muscles have developed a strong adaptive capacity. Particularly, they have a remarkable regenerative capacity, owing to their population of muscle stem cells. These cells are also named satellite cells based on their anatomical location between the basal lamina and the plasma membrane of myofibres [2,3]. Muscle stem cells are quiescent in a healthy resting muscle, but they are primed for activation. Following an injury, muscle stem cells quickly enter the cell cycle to become proliferative myoblasts that transiently expand the myogenic cell pool. Thereafter, myoblasts exit the cell cycle to differentiate and fuse to form multinucleated myotubes/myofibres. A proportion of myogenic cells also resist differentiation and self-renew to replenish the muscle stem cell pool [3–5]. Animal knockout models and genetic variants in humans leading to muscle stem cell exhaustion showed that these cells are absolutely required for muscle regeneration [6,7].

Skeletal muscle injury induces the coordinated accumulation of different cell types. Single-cell RNAseq and single-cell mass cytometry have identified between nine and 15 distinct cell populations in the resting skeletal muscle and during the different phases of regeneration [8–12]. After an injury, there is a rapid accumulation of immune cells (neutrophils, pro- and anti-inflammatory macrophages, natural killer cells, B- and T-cells) and changes in the proportion of non-immune cells (endothelial cells, smooth muscle cells, glial cells, tenocytes and fibro-adipogenic progenitors). These different cell types provide molecular cues to guide muscle stem cells through myogenesis [5]. For instance, during the course of muscle regeneration, there is a switch in macrophage phenotype from pro-inflammatory macrophages to anti-inflammatory macrophages. The former secretes cytokines that promote myoblast proliferation, while the latter releases factors stimulating myoblast differentiation and fusion [13,14]. In recent years, fibro-adipogenic progenitors (FAPs), a population of muscle-specific mesenchymal stromal cells, have emerged as master regulators of skeletal muscle regeneration [15–20]. This review aims at summarizing the recent knowledge on FAP cell characteristics, cellular interactions and roles in skeletal muscle under physiological and pathological conditions.

## 2. FAPs developmental origin and markers

The developmental origin of FAPs has been comprehensively reviewed recently [21,22]. The seminal work of Kardon and colleagues in the early 2000s identified a population of TCF7L2/TCF4+ (transcription factor 4; also known as transcription factor 7 like 2; TCF7L2) cells arising from the mesoderm lateral plate in the chick and mouse [23]. These cells do not express myogenic markers (Pax7) and do not form myotubes. However, functional experiments using a dominant-negative form of TCF7L2/TCF4 demonstrated that these cells are critical to set up a prepattern that plays an important role for determining myogenic cell differentiation in the limb [23]. Other markers expressed by connective tissue progenitors in the mouse embryo include, T-box transcription factors (TBX) 3/4/5, HOX11 and Odd skipped-related 1 (Osr1) [24–27]. Deletion of these genes leads to limb muscle patterning defects [24–26]. These genes are not universal markers for FAPs, but are rather expressed by different subpopulations that are spatially associated with specific regions of the limb [20,24,27,28].

**Figure 1.**
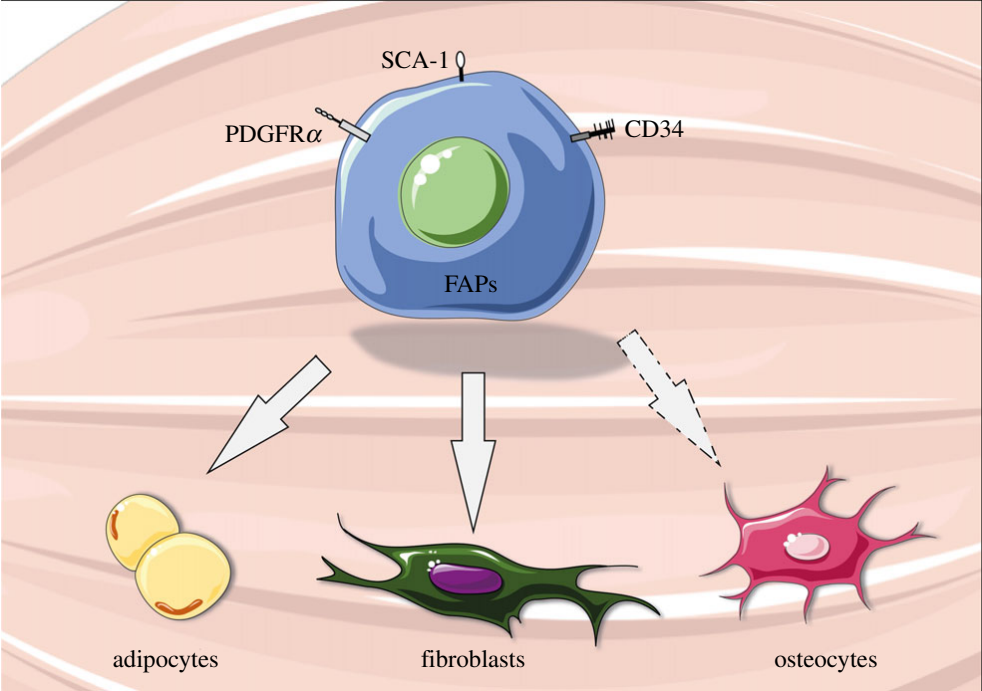
Schematic of fibro-adipogenic progenitors (FAPs). FAPs are muscle resident multipotent mesenchymal stem cells that can differentiate into adipocytes, fibroblasts or osteocytes (under specific conditions). They express key surface markers such as platelet-derived growth factor receptor α (PDGFRα), stem cell antigen-1 (Sca-1) and cluster of differentiation 34 (CD34). This figure was created with the Servier Medical Art service (https://smart.servier.com/), which is licensed under a Creative Commons Attribution 3.0 Unported License (https://creativecommons.org/licenses/by/3.0/).

In adults, FAPs are identified by the cell surface markers stem cell antigen-1 (Sca1 or Ly6A/E) and platelet-derived growth factor receptor α (PDGFRα) [15] (figure 1). They also express CD34, like other muscle resident cells such as muscle stem cells and endothelial cells [11,29–31], but not other myogenic markers such as integrin-α7 (itga7) and syndecan-4 [11]. Notably, in adult resting muscles, FAPs express low levels of Osr1, but it is re-expressed upon injury, suggesting that FAPs reactivate a developmental programme during muscle regeneration [32].

## 3. FAPs characteristics

**Figure 2.**
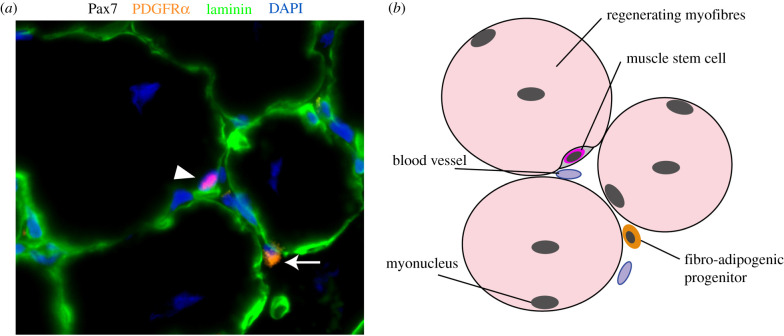
Fibro-adipogenic progenitors (FAPs) in regenerating skeletal muscle. (*a*) Immunofluorescence of Pax7 (red), PDGFRα (orange), laminin (green) and DAPI (nuclei, blue) on regenerating skeletal muscle section (14 days post-cardiotoxin injury). Muscle stem cell (Pax7+ cell in red; identified with white arrowhead) is visible in its niche under the basal membrane (laminin; green). FAP (PDGFRα+ cell in orange) is identified with a white arrow in the interstitial space. (*b*) Schematic of the location of muscle stem cell and FAP in skeletal muscle.

FAPs are located in the interstitial space of resting [21] or regenerating skeletal muscle (figure 2). They were initially described for their bi-potent ability to differentiate into fibrogenic or adipogenic cells, but not myogenic cells [15,17]. FAPs cultured *in vitro* can spontaneously differentiate into fibroblasts or adipocytes [15,17]. Their fibrogenic differentiation can be stimulated by adding transforming growth factor-β (TGF-β) to the medium, while their adipogenic differentiation can be promoted by a medium containing insulin, 3-isobutyl-1-methylxanthine and dexamethasone [33–35]. Lineage tracing experiments confirmed that this multipotent capacity is also observed *in vivo*. Experiments using PDGFRα-creERT *Rosa26*^EYFP^ reporter mice confirmed that perilipin+ adipocytes observed after cardiotoxin or glycerol-induced injuries originate from PDGFRα+ FAPs [36]. Another lineage tracing study using TCF7L2/TCF4^CreERT2/+^ *R26R*^mTmG/+^ reporter mice confirmed that cells expressing the fibroblast marker alpha-smooth muscle actin (αSMA) were originating from TCF7L2/TCF4+ FAPs [18].

Recent findings also indicate that FAPs can give rise to osteogenic or chondrogenic cells under specific culture conditions, such as bone morphogenic protein (BMP)-2, -7 or -9 administration [37–39]. Intramuscular transplantation of FAPs (CD31-CD45-Tie2+PDGFRα+Sca1+ cells) in combination with Matrigel and BMP2 demonstrated that these cells robustly contribute to cartilage and bone deposition *in vivo* [37]. Lineage tracing experiment using PDGFRα-creERT2-TdTomato mice confirmed that the vast majority of osteogenic cells came from FAPs in BMP2-induced heterotopic ossification *in vivo* [39]. These findings indicate that the multipotency of FAPs is broader than their name suggests.

## 4. FAPs in resting skeletal muscle

### 4.1. FAPs heterogeneity in muscle homeostasis

The emergence of single-Cell RNAseq analysis revealed cellular heterogeneity in the population of FAPs in resting muscles. A first study showed that FAPs sub-cluster in two different populations in non-injured muscles, one that is enriched for transcripts associated with ECM genes (e.g. *Col4a1*, *Col6a1*, *Lum*, *Sparcl1*, *Podn*, *Smoc2*, *Mgp* and *Bgn*) and the other one that express higher levels of genes involved in cell signalling pathways (e.g. *Sfrp4*, *Igfbp5*, *Sema3c*, *Dpp4*, *Tgfrb2* and *Wnt2*) [40]. Similar findings were observed in another study that has subdivided the FAPs population in non-injured muscles in Cxcl14-expressing FAPs that are enriched in ECM genes (e.g. *Col4a1*, *Col5a3*, *Col6a1*, *Smoc2* and *Lum*) and the Dpp4-expressing FAPs that are enriched in genes involved in different biological processes and signalling pathways (e.g. *Igfbp5*, *Igfbp6*, *Wnt2*, tnfaip6 and *Sema3c*) [9]. Furthermore, another study distinguished two cell subpopulations of FAPs based on the expression of *Tek* (gene encoding for Tie2) and *Vcam1* [41]. Tek+ FAPs are the predominant subpopulation in non-injured muscle and they preferentially express genes implicated in Wnt and BMP signalling (e.g. *Wnt5a*, *Wnt11*, *Bmp4* and *Bmp6*). *Vcam1*+ FAPs represent a smaller subset in non-injured muscle (that becomes the predominant subpopulation in response to acute injury) and display a pro-fibrotic gene signature (e.g. *Acta2*, *Adam12*, *Lox* and *Timp1*) [41]. Another recent study using unbiased scRNA-seq from healthy skeletal muscle also observed that *Tek* and *Vcam* expression are segregated in two different clusters in the FAPs population [42]. Moreover, this paper also demonstrated that the *Vcam1*+ population can be further subdivided to generate a third cellular subset characterized by the expression of thrombospondin-4 (*Thbs4*) and fibulin-7 (*Fbln7*) that is enriched in ECM organization and metallopeptidase activity genes [42]. Overall, while some discrepancies exist between the findings described in these independent studies, which could be attributable to different cell isolation/purification techniques and/or bioinformatic analyses, these scRNA-seq experiments reveal the cellular heterogeneity of FAPs and pinpoint common gene signatures of the different cellular subsets across the datasets.

### 4.2. Role of FAPs in the regulation of muscle homeostasis

FAPs are one of the predominant mononuclear cell population in non-injured muscle. These cells are quiescent in non-injured muscle, which is regulated, at least in part, by their expression of *hypermethylated in cancer-1* (Hic1) [40]. Deletion of this factor leads to spontaneous cell cycle entry and expansion of the FAPs population [40]. FAPs are required for homeostatic maintenance of skeletal muscle in steady-state conditions [16,17,43]. Depletion of FAPs induced by diphtheria toxin (DTX) administration to transgenic mice containing the *Fap* gene (fibroblast-activation protein-a) with the insertion of the DTX receptor led to a reduction of muscle mass and myofibre size after three weeks [43]. Similarly, FAPs depletion induced by tamoxifen injection to PDGFRα.creER-DTX mice induces muscle atrophy and reduces muscle force, which can last for months, even in the absence of injury [16,44]. Depletion of FAPs does not affect the number of muscle stem cells in the first few weeks [44], but a reduction in the number of muscle stem cells is noted nine months later (other cell types such as endothelial cells and haematopoietic cells were not affected) [16]. These results indicate that FAPs are required for the long-term homeostatic maintenance of the muscle stem cell pool and myofibre growth. As FAPs do not possess myogenic properties *per se*, these effects are mediated by molecular crosstalk with muscle cells.

FAPs provide a supportive environment for myogenic cells as they are the main source of extracellular matrix components, such as collagens (e.g. Col6a1, Col5a1), laminin (e.g. lama2, lamb1) and fibronectin (Fn1) [45]. These extracellular matrix proteins constitute the muscle stem cell niche and play a crucial role in their self-renewal [46–49]. FAPs also express cytokines and growth factors known to regulate myogenesis and muscle growth. It was shown *in vivo* that FAPs specifically express growth differentiation factor 10 (GDF10; also known as bone morphogenetic protein 3b, Bmp3b), a member of the TGF-β superfamily, that stimulates myotube hypertrophy *in vitro* (but not myoblast differentiation/fusion) by activating the Smad1/-5/-8 and Akt pathways [44]. Deletion of this factor in Bmp3b-knockout mice induces muscle atrophy [44]. Other *in vitro* experiments showed that FAPs are a major source of many trophic factors, such as interleukin-6 (IL-6), IL-10 and follistatin, among others, that promote myogenesis and muscle growth [15,50–53]. The exact cocktail of cytokines secreted by FAPs and their contribution in homeostatic condition remains elusive; however, the impact of these paracrine factors on skeletal muscle regeneration has been more extensively studied, and will be discussed hereafter.

## 5. FAPs in skeletal muscle injury/regeneration

### 5.1. FAPs dynamics in regenerating skeletal muscle

Upon acute muscle injury, FAPs rapidly enter cell cycle. The rise in BrDU^+^ cells happens faster in FAPs than in the muscle stem cell population, which leads to an increase in the FAPs/muscle stem cell ratio during the first few days after an injury [10,15]. The total number of FAPs peaks around 3–4 days post-injury, depending on the type and severity of injury [36,54]. Thereafter, there is a strong increase in cellular apoptosis and the number of FAPs gradually returns to basal level [54]. Conditional ablation experiments revealed the importance of FAPs during muscle regeneration. Depletion of FAPs induced by tamoxifen injection in PDGFRα.creER-DTX mice does not induce myofibre necrosis in non-injured mice [44], but it prolonged necrosis and led to significant regeneration deficit after acute muscle injury (BaCl_2_ injection) [16]. Transplantation of FAPs into the injured muscle of ablated mice rescued muscle regenerative capacity, confirming the crucial role of FAPs in muscle regeneration.

Single-cell RNAseq analysis revealed a dynamic and heterogeneous cluster of FAPs during regeneration [8,9]. At 2 days post-injury, activated FAPs express high levels of chemokines (e.g. *Ccl7*, *Cxcl5* and *Ccl2*) known to play a role in monocyte and neutrophil recruitment [8,9]. At this time point, there is also the emergence of a Tie2^low^ Vcam1^high^ subpopulation of FAPs expressing a pro-fibrotic gene signature [41]. At 5 days post-injury FAPs express higher levels of ECM genes such as *Col3a1*, *Col8a1*, *Dcn* and *Fn2*. After 7–10 days post-injury the expression profile of FAPs gradually returns to that observed in non-injured muscles (re-expression of markers such as CD34 and Sprouty RTK signalling antagonist 1 (Spry1)). However, at 21 days post-injury, the majority of FAPs still express high levels of Osr1, while this factor is weakly expressed in non-injured muscle, suggesting that these cells did not entirely return to quiescence at that point. Notably, Osr1+ FAPs can be divided in two subgroups, the dipeptidyl peptidase-4 (Dpp4) positive and the chemokine (C–X–C motif) ligand 4 (Cxcl4) positive cell populations indicating that they are re-acquiring cellular heterogeneity similar to what is observed in non-injured muscles [9,40].

### 5.2. Intrinsic regulation of FAPs in regenerating skeletal muscle

The accumulation of FAPs during muscle regeneration is tightly regulated. Yet, the intrinsic mechanisms controlling these dynamic changes are poorly characterized. One pioneer study showed that FAPs express different transcriptional variants of PDGFRα [55]. One of these variants contains a truncated kinase domain; and acts as a decoy receptor to inhibit PDGF signalling. Inhibition of this intronic polyadenylated variant increases FAPs proliferation and fibrogenic gene expression. The ratio of this intronic variant increases during muscle regeneration suggesting an intrinsic regulatory mechanism for FAPs to limit their expansion and activity. Another study showed that FAPs become ciliated upon muscle injury [36]. FAPs cilia represses Hedgehog signalling, a pathway that plays a crucial role to inhibit adipogenesis [36,56]. Consistently, deletion of FAPs cilia over-activates Hedgehog signalling and prevents adipogenesis following glycerol-induced muscle injury [36]. Another study indicated that retinoic acid receptor signalling plays an important role in the intrinsic regulation of FAPs number and cell fate decision [57]. Treatment of FAPs with retinoic acid promotes their proliferation *in vitro* and inhibits their adipogenic and fibrogenic differentiation both *in vitro* and *in vivo* (in obese mice fed with high-fat diet) [57]. Inversely, loss of retinoic acid receptor signalling specifically in FAPs promotes their adipogenic differentiation *in vitro* and *in vivo*; and it decreases cell apoptosis and delays their clearance following injury *in vivo* [57].

There is limited knowledge on the intrinsic mechanisms regulating FAPs during myogenesis; however, there is strong evidence that extrinsic factors play crucial roles in the regulation of FAPs behaviour. A transplantation experiment showed that FAPs isolated from cardiotoxin-injured muscles (which display virtually no adipogenesis) become adipocytes when transplanted into glycerol-injured muscles [17]. Conversely, FAPs isolated from glycerol-injured muscles do not become adipocytes when transplanted into cardiotoxin-injured muscles. These results suggest that the cell fate decision of FAPs is not regulated in a cell-autonomous manner, but rather by the extrinsic environment. The impact of inflammatory cells, myogenic cells and other muscle-resident cell types on FAPs will be further discussed hereafter.

## 6. FAPs cellular and molecular crosstalk in regenerating skeletal muscle

### 6.1. Interactions of FAPs with inflammatory cells

**Figure 3.**
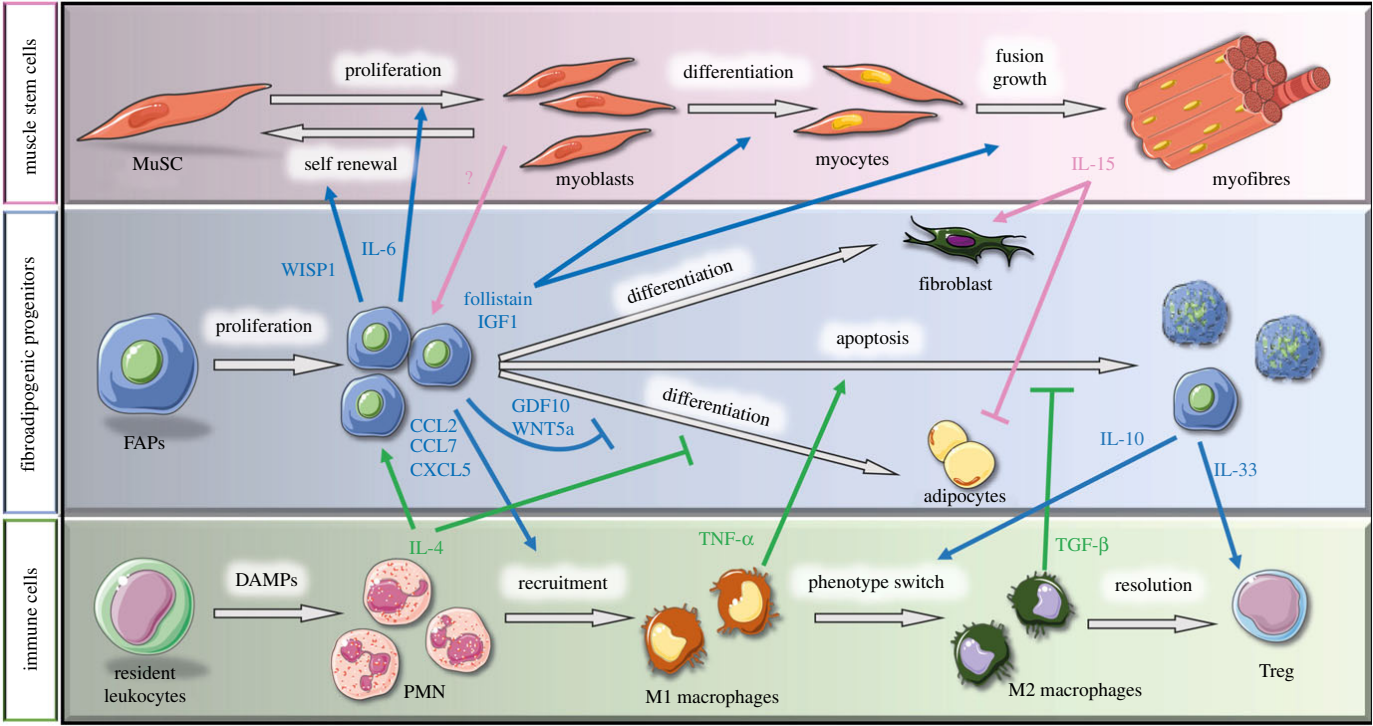
Cellular interactions during muscle regeneration. Schematic of the known interactions between muscle stem cells (MuSC, pink background), fibro-adipogenic progenitors (FAPs, blue background) and immune cells (green background) throughout muscle regeneration. The blue arrows represent molecules secreted by FAPs that act on other cell types, whereas pink and green arrows indicate paracrine factors secreted by muscle stem cells and immune cells, respectively, that affect FAPs. IL, interleukin; WISP1, Wnt1-inducible-signalling pathway protein 1; IGF1, insulin growth factor-1; GDF10, growth differentiation factor 10; TNF-α, tumour necrosis factor alpha; TGF-β, transforming growth factor-β; DAMPs, damage-associated molecular pattern; CCL, C–C motif chemokine ligand; CXCL5, C–X–C motif chemokine ligand 5, PMN, polymorphonuclear cells. This figure was created with the Servier Medical Art service (https://smart.servier.com/), which is licensed under a Creative Commons Attribution 3.0 Unported License (https://creativecommons.org/licenses/by/3.0/).

Skeletal muscle injury induces a rapid inflammatory response characterized by the subsequent accumulation of polymorphonuclear leucocytes, monocytes/macrophages and lymphocytes. The peak in inflammatory cell density coincides with the one of FAPs. Analysis of the interactome network from single-cell RNAseq data revealed that FAPs have strong receptor–ligand interactions with immune cells, especially polymorphonuclear cells and monocytes [9]. As mentioned above, in the early phase of muscle regeneration, FAPs express high levels of chemokines (e.g. *Ccl7*, *Cxcl5* and *Ccl2*) and immunomodulatory cytokines that regulate the accumulation and the function of inflammatory cells such as monocytes and neutrophils (figure 3) [8,9]. Inducible depletion of FAPs by tamoxifen injection in PDGFRα.creER-DTX mice followed by muscle injury is associated with a reduction in the accumulation of CD45+ haematopoietic cells during the acute phase of muscle regeneration [16,44]. Moreover, the transcriptional profile of a subset of FAPs during muscle regeneration is enriched in canonical pathways related to dendritic cell maturation, suggesting that they might play a role in the phenotypic switch of macrophages [41]. Particularly, activated FAPs upregulate their expression of IL-10 upon muscle damage [51]. This cytokine is a central effector that triggers the change in macrophage subsets toward their anti-inflammatory phenotype, which in turn secrete factors that promote the differentiation and fusion of myogenic cells [13,14,58].

Evidence also supports a role of FAPs in the modulation of regulatory T-cells (Treg) function. These cells peak late during muscle regeneration (4–7 days post-injury) and their numbers remain elevated for weeks after the injury. In skeletal muscles, FAPs are the main cellular source of IL-33, a member of the IL-1 family that has a potent immunomodulatory effect on Treg [59,60]. This alarmine is rapidly released upon muscle damage and stimulates the proliferation of Treg *in vitro* and *in vivo* [60]. Skeletal muscle injury in *Il1rl1*-null mice (IL-33 receptor) results in reduced accumulation of Treg during muscle regeneration and smaller size of newly formed myofibres [60]. Altogether, these findings suggest that FAPs regulate the accumulation and the function of immune cells, which in turn affect myogenesis.

The interactions between FAPs and immune cells are bilateral (figure 3). Different inflammatory cells were shown to play a key role in the regulation of FAPs accumulation, activity, cell fate decision and clearance. After an injury, the first cells to infiltrate the tissue are polymorphonuclear leucocytes. They enter the injured muscle as soon as 2 h post-injury and their numbers are peaking approximately 24 h post-injury, and rapidly decline afterward [61,62]. Among the polymorphonuclear cells, eosinophils were shown to play a central role in the regulation of FAPs [63]. Eosinophils are the main cellular source of IL-4 during muscle regeneration, and the IL-4 receptor (IL-4Rα) is highly expressed in FAPs. Lack of IL-4 signalling in Il4/Il13^−/−^ mice and Il4rα^−/−^ mice reduces FAPs proliferation (no effect on myogenic cell proliferation) and impairs muscle regeneration [63]. IL-4 also prevents FAPs differentiation into adipocytes *in vitro*, by repressing the expression of genes normally involved in adipogenesis (*Pparg*, *Lep*, *Fabp4*, *Acaca*, *Cd36* and *Dgat2*) [63]. Moreover, FAPs showed a strong phagocytosis capacity *in vitro* and *in vivo*, which is impaired in mice lacking type 2 innate immunity (Il4/Il13^−/−^, Il4rα^−/−^) and in eosinophil-deficient mice (ΔdblGATA mice) leading to lack of clearance of necrotic debris in injured muscles. Administration of IL-4 *in vivo* reduced the number of necrotic cells in injured Il4/Il13^−/−^ mice, suggesting that it rescues the phagocytotic capacity of FAPs (although a reduction in muscle degeneration through other mechanisms cannot be excluded) [63]. Altogether, these results indicate that the production of IL-4 by eosinophils regulates FAPs proliferation, phagocytosis and cell fate decision.

Monocytes/macrophages also play a key role in the regulation of FAPs. Clodronate-induced depletion of macrophages prior to muscle injury leads to a strong increase in fatty tissue infiltration [34]. Similarly, muscle injury in Ccr2-knockout mice, in which monocytes are not able to infiltrate the injured tissue, leads to the prolonged persistence of FAPs in the regenerating muscle associated with fibrosis deposition [54]. The impact of the depletion of monocytes/macrophages on the accumulation of FAPs is mediated by changes in cell apoptosis and not in cell proliferation. TNF-α, which is highly secreted by pro-inflammatory macrophages during acute injury, was shown to be the effector of FAPs apoptosis [54]. Another pro-inflammatory factor secreted by pro-inflammatory macrophages, IL-1α/β, was also shown to inhibit adipogenic differentiation of FAPs [64]. As the regeneration process progresses, there is a switch in macrophage phenotype towards anti-inflammatory macrophages, which secrete higher levels of TGF-β. This growth factor competes with TNF-α and promotes the survival of FAPs. TGF-β was also shown to downregulate the expression of the FAPs markers PDGFRα and TCF7L2/TCF4, and their downstream signalling pathways [65,66]. TGF-β influences the cell fate decision of FAPs by enhancing their survival, promoting their proliferation, inhibiting their adipogenic differentiation and favouring their differentiation into myofibroblasts [54,67,68]. *In vitro* experiments showed that IL-1β-polarized pro-inflammatory macrophages (expressing high levels of IL-6 and TNF-α) inhibits FAPs differentiation towards adipogenic cells, while IL-4-polarized anti-inflammatory macrophages promote the formation of adipocytes [69]. Altogether, these results indicate that the switch in macrophage phenotype needs to be fine-tuned to avoid excessive accumulation of FAPs and fibrotic tissue deposition, and to promote return to homeostasis and long-term maintenance of the cellular reserve.

### 6.2. Interaction of FAPs with myogenic cells

The formation of new muscle tissue during muscle regeneration needs to be closely coordinated with the remodelling of the ECM. Accordingly, FAPs maintain a close proximity with myofibres in resting muscle and during muscle regeneration (figure 2) [15,17]. After an injury, FAPs quickly enter the cell cycle (BrdU+ or EdU+ cells) and the ratio FAPs/MuSC increases during the first few days, suggesting that they may act upstream and help orchestrate the myogenesis process [10,15]. Cardiotoxin injury in FAPs-depleted muscles (PDGFRα.creER-DTX mice) is associated with a strong decline in the number of myogenic cells at an early time point (3 days post-injury), and a reduction in the size of the newly formed fibres at a later time point (14 days post-injury) [16,44]. Similar impairment in myogenic cell pool expansion and muscle regeneration is observed when mice are treated with the tyrosine kinase inhibitor Nilotinib (PDGFR inhibitor), which induces FAPs apoptosis and reduces the number of FAPs in the injured muscle [70,71]. *In vitro* co-culture experiments showed that FAPs preferentially promote the differentiation of myogenic cells and their fusion into multinucleated myotubes [15,17]. Gene expression analysis confirmed that FAPs reduce the expression of early myogenesis markers (e.g. *Pax3*, *Pax7*) and promote the expression of late myogenic differentiation markers (e.g. *MyoD*, *Myog*). Moreover, TCF7L2/TCF4+ cells (likely FAPs) [65] also regulate muscle fibre type and maturation. *In vitro*, transwell co-culture experiments of TCF7L2/TCF4+ FAPs and myogenic cells showed that paracrine factors secreted by FAPs enhance fusion index and slow MyHC expression. *In vivo*, the deletion of TCF7L2/TCF4+ using different transgenic mouse models increases the developmental myosin heavy chain (MyHC) expression and decreases the expression of slow MyHC in neonatal mice [20].

The mechanisms by which FAPs orchestrate muscle stem cell activity during myogenesis is under intense investigation in the field. FAPs are a predominant source of paracrine factors during muscle regeneration (figure 3) [15,17]. The main molecular components of the secretome of FAPs were comprehensively summarized in two recent reviews [53,72]. Briefly, activated FAPs express high levels of cytokines such as IL-6. This cytokine has been described as a myokine (i.e. secreted by myofibres in response to exercise); however, its expression in regenerating muscle is roughly 10-fold higher in FAPs than in myogenic progenitor cells [15,17]. IL-6 promotes muscle stem cell proliferation and myogenic progression [73,74]. Activated FAPs also strongly upregulate the expression of WNT1-inducible-signalling pathway protein 1 (WISP1) [75]. WISP1 promotes muscle stem cell expansion and myogenic commitment via asymmetric cell division [75]. FAPs also express high levels of IGF-1, which activates the Akt pathway that controls both protein synthesis and degradation, resulting in skeletal muscle growth [15,17,76]. In accordance with a role of FAPs in late stages of myogenesis, activated FAPs are the main mononuclear cell source of follistatin during acute muscle regeneration post-injury [50,77]. This activin-binding protein is an antagonist of the muscle growth inhibitor myostatin. FAPs-secreted follistatin also promotes myoblasts fusion into multinucleated myotubes [77,78]. Overall, FAPs secrete a cocktail of cytokines that have an impact on myogenic cells throughout the different stages of myogenesis.

Myogenic cells also contribute to the regulation of FAPs during muscle regeneration (figure 3). *In vivo*, the deletion of muscle stem cells using the Pax7.CreERT2-DTX mice model impaired the expansion of FAPs (TCF7L2/TCF4-expressing cells) during the early phase of muscle regeneration (5 days post-injury) but led to their prolonged accumulation and fibrosis deposition at a later time point (28 days post-injury) [18]. Muscle stem cells secrete exosomes containing miRNA, such as miR206, which inhibit collagen production by TCF7L2/TCF4+ FAPs [79]. *In vitro* experiments showed that myoblast conditioned medium promotes FAPs proliferation. Satellite cells were shown to secrete betabellulin and epidermal growth factor (EGF), two ligands of the EGF receptor (EGFR), which stimulate FAPs proliferation *in vitro* [64]. Notably, contrary to myoblast conditioned medium, myotube-conditioned medium inhibits the expression of adipogenesis genes and upregulates the expression of fibrogenesis genes, suggesting that factors secreted during late stage of myogenesis could impact on the cell fate decision of FAPs and ECM remodelling [17,80]. Other studies have suggested that inhibition of adipogenesis by differentiated myoblasts is mediated by direct contact rather than secreted factors [17,34]. The binding of delta-like ligand (DLL) expressed on the myogenic cells to the Notch receptor on the FAPs would lead to a Notch-dependent inhibition of adipogenic gene expression [34].

Among the myokines secreted by muscle cells after injury, IL-15 was shown to influence FAPs behaviour. IL-15 administration stimulates FAPs proliferation *in vitro* and *in vivo* [81]. This pro-proliferative effect is mediated trough Jak-Stat pathway, and the administration of the Jak inhibitor SAR-20347 decreased FAPs proliferation and prevented fibrosis deposition post-injury. IL-15 also influences cell fate decision of FAPs, and prevents their differentiation into adipocytes *in vitro* and *in vivo* [81–83]. This IL-15-mediated decrease in adipogenesis is associated with an upregulation of desert hedgehog (DHH) signalling pathway and its downstream effector tissue inhibitor of metalloproteinase 3 (Timp3). This pathway is regulated by primary cilia in FAPs, which has been demonstrated to repress FAPs adipogenic differentiation [36]. Conversely, IL-15 treatment increases ECM gene expression and collagen deposition in injured skeletal muscle [81]. These findings suggest that IL-15 controls the cell fate decision of FAPs to preferentially favour fibrogenesis over adipogenesis.

### 6.3. Interaction of FAPs with other muscle-resident cell types

Interstitial stromal cells can also have auto-regulatory effects on FAPs. These cells were identified by single-cell RNAseq analysis based on the expression of interstitial stromal cell markers such as the FAPs markers *Sca1* and *PDGFRa*, the pericytes or mesoangioblasts marker *Alpl* (Alkaline phosphatase), and the PW1+ interstitial cells (PICs) marker *Peg3* (paternally expressed gene 3). These interstitial stromal cells were subdivided in three subsets [42]. These subpopulations express high levels of adipogenesis genes, except one cluster expressing CD142 [42]. The CD142+ interstitial stromal cells are able to differentiate into fibrogenic cells but not adipogenic cells. Remarkably, when co-culture together, the CD142+ interstitial stromal cells inhibit the adipogenic differentiation of CD142− interstitial stromal cells. The secretion of high levels of GDF10 by these adipo-regulatory cells is at least partially responsible for the suppression of adipogenesis. FAPs are also the main mononuclear cell source of Wnt signalling protein in skeletal muscles [78]. The secretion of Wnt5a by FAPs mediate an autocrine response that activates β-catenin signalling and blocks adipogenesis. These results indicate that the different subsets of FAPs could regulate their own cell fate decision.

Schwann cells also participate in the regulation of FAPs cell fate [36]. Single-cell transcriptomics of injured muscles indicate that Schwann cells are the main source of Dhh signalling during muscle regeneration [8,9]. Notably, Schwann cells express Dhh following cardiotoxin injection, a model of injury that is devoid of adipose tissue deposition; however, Schwann cells do not express Dhh in glycerol-induced injury, which causes severe adipogenesis [36]. Dhh activates the expression of cilia-dependent Hedgehog target genes, particularly Timp3, which represses adipogenesis through inhibition of matrix metalloproteinase 14 (Mmp14).

Analysis of the putative interactome also indicates strong ligand–receptor interactions between endothelial cells and FAPs [9], which is coherent with the perivascular localization of FAPs [15,17,19,40]. Deletion of PDGFRα+ cells by tamoxifen and DTX administration to *Pdgfra-MerCreMer*/inducible-DTX receptor mice did not induce vascular tissue disruption in the short term in the absence of injury [19]. However, after hindlimb ischaemia, impairments in vessel size, organization and permeability were observed following PDGFRα cell ablation [19]. FAPs are a source of vascular endothelial growth factor (VEGF), which is known to promote postnatal angiogenesis [84]. Yet, the exact interplay between FAPs and endothelial cells in muscle homeostasis and regeneration needs to be further explored [72].

Overall, FAPs are central regulators of muscle homeostasis and regeneration. Their complex molecular and cellular interactions with inflammatory cells, myogenic cells and other resident cell types play a critical role in the coordination of skeletal muscle response to injury. These intrinsic and extrinsic regulatory mechanisms control the activation, proliferation, cell fate decision and clearance of FAPs to avoid the prolonged or excessive accumulation of fibrofatty tissue. However, this delicate balance is perturbed in different pathological conditions in which FAPs contribute to disease progression. The impact of FAPs in muscular dystrophies and ageing will be discussed in this next section.

## 7. FAPs in muscular dystrophies

### 7.1. Impact of FAPs in the pathogenesis of Duchenne muscular dystrophy

Loss of muscle mass and replacement with fibrous and fatty tissue is a hallmark of many muscular diseases. Considering the intrinsic ability of FAPs to differentiate into adipogenic or fibrogenic cells, they are likely to be the main effector of these pathological processes. Accumulating evidence places FAPs on the front line of several muscular diseases.

Among myopathies, DMD is the most studied. This pathology is caused by a mutation in the *DMD* gene which encodes for the dystrophin protein [85,86]. The dystrophin–dystroglycan complex connects the actin cytoskeleton of the muscle fibres to the extracellular matrix, and acts as a force transduction system during muscle contraction [87]. Lack of dystrophin induces muscle fibre fragility and cycle of degeneration and regeneration leading to muscle wasting, chronic inflammation and fibrosis deposition [3,88,89]. Endomysial fibrosis and fat accumulation are among the only parameters correlated with poor motor outcomes in DMD [90–92]. Therefore, a lot of effort has been invested to characterize the cells responsible for the fibrofatty accumulation and the underlying mechanisms driving fibrofatty accumulation in order to develop new therapeutic avenues [54,93].

By contrast to acute injury, which induces a transient expansion of the FAPs population, dystrophic muscles experience a persistent increase in the number of FAPs [10,35,54,94]. In dystrophin-null *mdx* mice, the vast majority of collagen-expressing cells (identified by Col1a1-GFP reporter) are PDGFRα+ and Sca1+ cells, suggesting that FAPs are the main source of fibrosis [95].

### 7.2. FAPs heterogeneity in Duchenne muscular dystrophy

An increase in cellular heterogeneity is observed in FAPs in dystrophic muscles. Two subpopulations of FAPs, the Sca1-low/CD34-low and Sca1-high/CD34-high subsets, which are predominantly found in non-injured and injured wild-type muscles, respectively, are both found alongside within *mdx* muscles [34]. The Sca1-high subpopulation proliferate faster than the Sca1-low subpopulation and are predisposed to differentiate into adipocytes [33]. Similarly, there is the emergence of a subpopulation of PDGFRα-low FAPs expressing high levels of fibroblast associated genes (*TGFb1*, *Col1a1* and *CTGF*) in the skeletal muscles of *mdx* mice [66]. FAPs can also be subdivided in Vcam1− and Vcam1+ subpopulations in dystrophic muscles, the latter being absent from non-injured wild-type muscles [41]. This Vcam1+ subset has a pro-fibrotic transcriptional profile and has high proliferative state compared to other FAPs subpopulations [41]. Single-cell RNAseq analysis from different datasets suggests that there is an overlap between these different subsets, although it remains to be confirmed precisely [8,9]. Furthermore, there are also differences within the analogous subpopulations depending on the pathophysiological context. For example, Tie2-high subpopulation isolated from acute injured muscle displays changes in gene expression related to biological function such as muscle growth and dendritic cell maturation; however, these characteristics are not observed in Tie2-high subpopulation isolated from DMD muscle [41].

### 7.3. Regulatory network of FAPs in Duchenne muscular dystrophy

Considering the importance of the molecular and cellular crosstalk in the regulation of FAPs accumulation and cell fate decision, it is likely that the changes observed in FAPs behaviour are attributable to the degenerative microenvironment found in dystrophic muscles. Contrary to wild-type mice that display a coordinated response following injury, cardiotoxin-induced muscle injury in *mdx* mice does not induce the expansion of FAPs, which is associated with impairment in the myogenic cell pool growth and macrophage phenotype switching [10]. These findings suggest that the molecular signals and cellular interactions are perturbed in dystrophic muscles, which consequently deregulate the accumulation and clearance of FAPs.

Among the different molecules upregulated in DMD, TGF-β is a key regulator of FAPs functioning. A positive correlation between TGF-β levels, FAPs content and fibrosis has been observed in different muscle injuries and diseases such as denervation [66], glycerol-induced injury [66], amyotrophic lateral sclerosis (hSOD1^G93A^ mice) [96] and DMD (*mdx* mice and DMD patients) [97–99]. In DMD, the chronic presence of macrophages expressing a hybrid phenotype (secreting both TNF-α and TGF-β) leads to conflicting signals to FAPs that fail to induce apoptosis [54]. Moreover, FAPs are the main producers of TGF-β-activating enzymes such as BMP1 and MMP14 that activate the latent-TGF-β secreted by macrophages [100]. High levels of TGF-β reduce the expression of TCF7L2/TCF4, as well as PDGFRα and its target genes, leading to the emergence of a TCF7L2/TCF4-low PDGFRα-low subpopulation of FAPs exhibiting a pro-fibrotic profile [65,66,101]. Corroborating with those findings, D2-*mdx* mice, which carry a genetic variant in the latent transforming growth factor binding protein 4 (LTBP4) that reduces its capacity to sequester TGF-β (consequently increasing the levels of active TGF-β), display increased FAPs accumulation and fibro-calcification deposition [101]. Noteworthy, chronic exposure to high levels of TGF-β in dystrophic muscles induces changes in a subset of endothelial cells and satellite cells that lose their cell identity and acquire a mesenchymal-like phenotype [97]. These cells acquire the expression of PDGFRα, produce higher levels of fibrogenic genes (e.g. Col1a1, Fn1 and Acta2) and gain the capacity to transdifferentiate into fibroblasts *in vitro*, suggesting that cell types other than FAPs could also contribute to fibrogenesis in DMD [97].

Evidence also indicates that the molecular signals provided by myogenic cells to FAPs through direct contact or by secretion of paracrine factors are also perturbed in DMD. Conditioned medium from myogenic cells isolated from DMD patients failed to promote FAPs proliferation and fibrogenic differentiation [80]. Moreover, while in healthy regenerating muscle the expression of the Notch ligand on the cell surface of differentiated myoblasts/myotubes inhibits adipogenic differentiation of FAPs by direct cellular contact, FAPs from *mdx* mice are insensitive to NOTCH-induced adipogenic inhibition [34].

Cilia-mediated signalling could also regulate FAPs adipogenic cell fate decision in dystrophic muscles. Although there is not a significant increase in the proportion of ciliated FAPs in *mdx* mice, the deletion of cilia specifically in FAPs reduces the number of adipocytes and enhances muscle regeneration in dystrophic muscles [36]. The impact of FAPs cilia on the regulation of adipogenesis is non-cell autonomous, suggesting a lack in hedgehog protein secreted from neighbouring cells.

Impairment in the secretion of autocrine factors by FAPs in dystrophic muscles could also have an impact on their cell fate decision. Analysis from bulk RNAseq datasets revealed downregulation of WNT ligands and receptors in FAPs from *mdx* mice, which could affect their cell fate considering the importance of the WNT5/GSK3/β-catenin pathway in the regulation of adipogenesis [78]. Consistently, treatment of *mdx* FAPs with WNT5a prevents β-catenin downregulation, which inhibits PPARγ expression and adipogenesis.

Overall, intrinsic and extrinsic factors contribute to the persistence of FAPs and to the dysregulation of their activity and cell fate decision in DMD.

### 7.4. FAPs in other muscular dystrophies

Apart from their role in DMD, FAPs are also implicated in the pathogenesis of many other muscular diseases. In limb girdle muscular dystrophy type 2 (LGMD2B), adipogenic replacement in dysferlin-deficient skeletal muscle is correlated with the severity of the disease [102]. Sustained muscle injury induces the extracellular release of annexin-A2, which causes FAPs accumulation and adipogenic differentiation [102]. In a model of LGMD2E (sarcoglycan-β deficient mice), it was shown that chronic muscle degeneration impairs the cellular heterogeneity of the different subsets of interstitial stromal cells (analogous to FAPs). Particularly, there is a reduction in the population of CD142+ interstitial stromal cells, which act as adipo-regulatory cells that inhibits adipocyte differentiation through GDF10 secretion [42]. Other congenital muscular dystrophies such as merosin-deficient congenital muscular dystrophy type 1A (LAMA2 deficiency) or Ullrich congenital muscular dystrophy (mutations in *COL6A1*, *2* or *3*) are also characterized by muscle degeneration and fibrosis accumulation [103–105]. LAMA2 deficiency in skeletal muscle is associated with an over-activation of the TGF-β pathway and fibrotic tissue deposition that correlates with the severity of the disease [106–108]. Losartan treatment (an angiotensin II receptor blocker) in LAMA2-mutant mice reduces TGF-β expression and fibrosis deposition [109]. Likewise, COL6A1 myopathies are characterized by an excessive accumulation of PDGFRα+ cells and fibrosis in mouse model and in patients affected by Ullrich congenital muscular dystrophy [105]. The exact contribution of FAPs to the pathogenesis of these diseases remains to be determined.

## 8. Therapeutic avenues targeting FAPs in muscular dystrophies

### 8.1. Glucocorticoids

**Figure 4.**
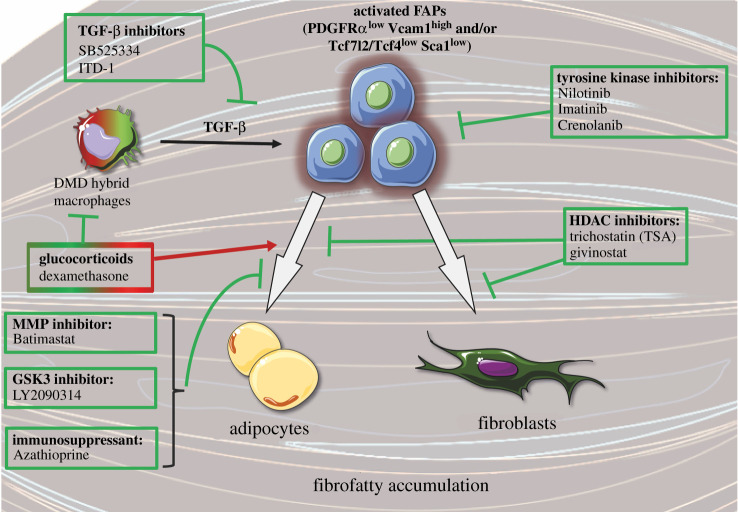
Molecular approaches targeting fibro-adipogenic progenitors in muscular dystrophies. Schematic of different drugs tested to inhibit fibro-adipogenic progenitor (FAP) activity, proliferation and/or their differentiation into adipocytes or fibroblasts. Arrows and T-shaped lines indicate stimulatory and inhibitory effects, respectively. DMD, Duchenne muscular dystrophy; HDAC, histone deacetylase; Vcam1, vascular cell adhesion molecule 1; PDGFRα, platelet-derived growth factor receptor α; TCF7L2/TCF4, transcription factor 4; Sca1, stem cells antigen-1; TGF-β, transforming growth factor-β; MMP, matrix metalloproteinase; GSK3, glycogen synthase kinase-3. This figure was created with the Servier Medical Art service (https://smart.servier.com/), which is licensed under a Creative Commons Attribution 3.0 Unported License (https://creativecommons.org/licenses/by/3.0/).

Glucocorticoids are the current gold-standard treatment for DMD. These drugs dampen inflammation and temporarily prevent the loss in muscle force and physical function [110]. However, glucocorticoids have numerous side effects. Glucocorticoids administration to fibroblasts or mesenchymal stromal cells *in vitro* increases the expression of adipocyte-associated gene (e.g. *Pparg*) and promotes adipocyte differentiation [111–113]. Inhibition of glucocorticoid receptor signalling using pharmacological antagonists or knockout models blocks the adipogenic differentiation of these cells *in vitro* [113]. *In vivo*, the administration of dexamethasone, a glucocorticoid, stimulates FAPs proliferation and adipogenic differentiation in injured muscle by inhibiting IL-4 expression [111]. A similar increase in adipogenesis was also observed *in vivo* following daily injection of prednisone, another glucocorticoid, in dystrophic mice [114]. Recent findings also suggest that glucocorticoids can have pro- or anti-adipogenic effects on FAPs depending on the type of glucocorticoids used and on the culture conditions [115]. For instance, glucocorticoids such as halcinonide and clobetasol have no effect on adipogenesis, while budesonide has anti-adipogenic effect *in vitro* [115]. This anti-adipogenic effect of budesonide was only observed when FAPs were actively proliferating *in vitro*. Conversely, budesonide has a pro-adipogenic effect when FAPs are confluent and are incubated in adipogenic induction medium [115]. Overall, while glucocorticoids preserve muscle function by reducing inflammation, there is room for improvement to develop therapeutic drugs targeting FAPs, directly or indirectly, to promote their beneficial effects (e.g. stimulation of myogenesis) and limit their harmful side effects (e.g. excessive fibrofatty accumulation) (figure 4).

### 8.2. TGF-β inhibitors

Considering the correlation between TGF-β levels, FAPs content and fibrosis, many studies have investigated the therapeutic potential of TGF-β inhibitors. Administration of the TGF-β inhibitor, SB525334, which blocks the ATP-binding site of TGFBR1 and inhibits the TGF-β induced signalling, on FAPs cultured *in vitro* prevented the decrease in PDGFRα levels induced by TGF-β [66]. Intramuscular injection of ITD-1, a small molecule enhancing TGFBR2 degradation thereby inhibiting TGF-β-induced signalling, reduced the accumulation of FAPs and muscle fibrosis in injured D2-*mdx* mice [101]. Inhibition of the TGF-β-activating enzymes MMP14 and BMP1 (with the compounds NSC-405020 and UK-383367, respectively) also reduced collagen deposition and increased myofibre size in a fibrotic model of *mdx* mice [100]. Similarly, inhibition of the TGFBR1 expression using antisense oligonucleotide intramuscular injection in mdx mice reduced the expression of fibrotic genes (e.g. *Col1a1*) and increased the expression of myogenic genes (e.g. *Myog*) [116]. Altogether, the different strategies used support the therapeutic potential of targeting TGF-β to inhibit the fibrogenic activity of FAPs and fibrosis deposition in dystrophic muscles.

### 8.3. GSK-3 inhibitors

Library drug screening of kinase inhibitors on FAPs isolated from *mdx* mice showed an enrichment in GSK3-targeting molecules having anti-fibrotic and/or anti-adipogenic effects [78]. Administration of the GSK3 inhibitor, LY2090314, represses FAPs adipogenesis in glycerol-injured muscle *in vivo*. Moreover, GSK3 inhibitor enhances the secretion of paracrine factors, particularly follistatin, that promotes myogenesis [78]. The same group used a similar drug screening strategy with a different small molecule library, and they identified azathioprine (AZA) as a promising therapeutic compound [117]. This immunosuppressant drug targets FAPs and blunts their adipogenic differentiation *in vitro*. AZA inhibits the insulin-mediated activation of the Akt-mTOR pathway in FAPs isolated from *mdx* mice.

### 8.4. Tyrosine kinase inhibitors

The tyrosin kinase inhibitors, nilotinib and imatinib, initially designed to target the BCR-ABL fusion oncogene in chronic myelogenous leukaemia, also showed significant anti-fibrosis capacity. Treatment of *mdx* mice with these compounds increases FAPs apoptosis, and reduces FAPs accumulation and fibrogenic differentiation leading to lower collagen deposition [54,118,119]. Nilotinib and imatinib are potent inhibitors of PDGFRα and can directly target FAPs to regulate their activity. Similarly, crenolanib, another PDGFRα/β inhibitor, was also shown to reduce ECM protein expression and fibrosis deposition in skeletal muscles of *mdx* mice [95]. These tyrosin kinase inhibitors could also indirectly regulate FAPs activity by acting on inflammatory cells. Imatinib treatment was shown to decrease macrophage infiltration in *mdx* mice [118,119]; while nilotinib was shown to block the anti-apoptotic effect of TGF-β secreted by macrophages and restore the pro-apoptotic capacity of TNF-α [54]. Furthermore, nilotinib also inhibits the phosphorylation of p38 induced by TGF-β administration to the C3H10T1/2 cell line (murine mesenchymal stromal cell line), suggesting that the effect of this drug on FAPs could also be mediated by off-target mechanisms [71,120].

### 8.5. Histone deacetylase inhibitors

Histone deacetylase (HDAC) inhibitors have also been investigated for the treatment of DMD, due to their ability to remove the repressive epigenetic marks and promote the expression of muscle-specific genes. Preclinical trials on *mdx* mice showed that HDAC inhibitors such as trichostatin A (TSA) or givinostat reduce fibrosis and promote muscle regeneration and function [121–123]. A phase 2 clinical trial also showed that givinostat induces a reduction in fibrosis in DMD boys [124]. The underlying mechanism responsible for these effects seems to be mediated by FAPs [77]. Treatment of FAPs isolated from *mdx* mice with TSA reduces their differentiation into adipocytes and promotes their myogenic capacity through secretion of paracrine factors such as follistatin. However, these effects are only observed in young *mdx* mice (1.5 months old), suggesting that long-term exposure to the detrimental microenvironment in aged *mdx* mice (12 months old) induces intrinsic cellular changes in FAPs [77,125]. Noteworthy, while TSA inhibits adipogenesis, it has also been shown to promote fibrogenic differentiation of FAPs [78].

### 8.6. Metalloproteinase inhibitors

Considering the role of metalloproteinases in ECM remodelling, the therapeutic potential of MMP inhibitors was also investigated in muscular dystrophies. The expression of many MMPs is upregulated in the skeletal muscle of *mdx* mice, while the tissue inhibitors of MMP (TIMP) are downregulated [126]. Administration of the MMP inhibitor batimastat in *mdx* mice decreases fibrosis accumulation and increases muscle function [126]. Treatment with batimastat also blocks adipogenic differentiation of FAPs *in vitro* [102]. It does not affect the accumulation of FAPs in the skeletal muscle of LGMD2B mice, but it reduced their adipogenic differentiation [102]. Moreover, as mentioned above, some MMPs, such as MMP14, can convert latent-TGF-β to its active form. Thus, MMP inhibition could also reduce fibrosis by dampening TGF-β expression [100].

### 8.7. *In vivo* validation

Although these studies do not represent an exhaustive list of the different drugs tested, they illustrate that there are different strategies aiming to target FAPs. Noteworthy, drug screening on FAPs *in vitro* is a useful method to identify potential targets; however, the therapeutic impact of these compounds does not often transfer into benefits *in vivo*. A recent study screened two libraries containing 722 compounds to determine their potential to block fibrogenesis [71]. The compounds were tested on FAPs expressing the enhanced green fluorescent protein (EGFP) under the *Collagen1a1* promotor. Only 21 compounds exerted a dose-dependent ability to reduce EGFP expression induced by TGF-β. Masitinib and sorafenib, two tyrosine kinase inhibitors as well as JQ1, a member of the bromodomain inhibitor family, were the most potent candidates. These lead compounds were tested *in vivo*, and they had mixed effects on muscle fibrosis in dystrophic mice. None of these drugs strongly and consistently reduced muscle fibrosis. Daily intra-peritoneal injections of masitinib for four weeks leads to a slight decrease in the total collagen content in the diaphragm, but its administration through osmotic minipump for eight weeks does not improve fibrosis deposition. Administration of JQ1 has no effect on the total collagen content of the diaphragm, but it reduces collagen deposition in both TA and gastrocnemius [71]. Noteworthy, the administration of JQ1 also induced muscle mass loss and myofibre atrophy. Different factors could explain the discrepancy between *in vitro* and *in vivo* results such as adverse effect on other cell types (e.g. leucocytes, myogenic cells and myofibres), wrong dosage or administration route. Therefore, the positive hits need to be carefully validated *in vivo* using standard operating procedures for preclinical studies on dystrophic animals. Nonetheless, these findings provide a proof-of-concept that targeting FAPs is a promising therapeutic strategy to mitigate muscular dystrophies.

## 9. FAPs and ageing

Ageing is characterized by a decline in several physiological functions. Particularly, ageing is associated with sarcopenia, a process characterized by a progressive and generalized loss of skeletal muscle mass and function leading to failure in the elderly [127]. It is well-characterized that, during the course of ageing, there is a reduction in the muscle stem cell pool and intrinsic cellular defects leading to impaired muscle regeneration [128–130]; even though, the contribution of these defects to the development of sarcopenia is still debated [131,132]. Defects in the secretion of paracrine factors by FAPs also contribute to this impaired regenerative response. FAPs are the main mononuclear cell source of IL-33, a cytokine associated with type 2 immunity; however, during ageing, there is a reduction in the production of this cytokine by FAPs leading to reduced accumulation of Treg and poor muscle repair [60]. Moreover, aged FAPs express lower levels of WISP, which plays an important role in the asymmetric division of muscle stem cells and muscle regeneration [75]. Aged FAPs also express lower levels of GDF10. *In vitro*, the addition of conditioned medium obtained from transgenic FAPs overexpressing GDF10 induces myotube hypertrophy to a higher level than conditioned medium harvested form WT or GDF10-knockout FAPs. The administration of GDF10 to aged mice reverses the loss of muscle mass and myofibre atrophy [44]. Supporting the importance of FAPs in the defect of myogenesis during ageing, the cellular transplantation of young FAPs in aged mice restores the myogenic commitment of muscle stem cells [75].

Muscle ageing is characterized by an increased amount of fibrotic tissue, suggesting impairment in the activity of FAPs [44,75]. It has been shown that during muscle ageing, there is a reduction in the number of FAPs and their proliferative capacity, as well as an increased predisposition to fibrogenic differentiation [44,75]. Different intrinsic cellular defects have been shown to contribute to these perturbations in FAPs activity during ageing. A reduction in the truncated PDGFRα variant that acts as a decoy receptor to inhibit PDGF signalling has been observed in aged FAPs [75]. Moreover, ageing is associated with an increase in cellular senescence (state of irreversible cell cycle arrest) [133,134]. In aged skeletal muscle, cellular senescence has been observed in muscle stem cells [129], but also in a population of interstitial cells [135]. Induction of cellular senescence in mesenchymal stromal cells *in vitro* blunted their fibrogenic and adipogenic differentiation ability. Moreover, the release of senescence-associated secretory phenotype factors by senescent mesenchymal stromal cells blocks the formation of myotubes *in vitro* [135]. Further studies are required to determine the contribution of FAPs cellular senescence to the pathogenesis observed in aged skeletal muscle.

The alterations in FAPs behaviour could be mediated by changes in the microenvironmental cues during muscle ageing. For instance, there are higher levels of the profibrotic factor TGF-β during ageing [136]. There are also lower levels of IL-15, a cytokine that plays a key role in the regulation of FAPs proliferation and cell fate decision [81,82]. Changes in the secretion of paracrine factors by neighbouring cells also contribute to this dysfunction. The conditioned medium of myogenic cells isolated from young individuals increases FAPs proliferation and inhibits adipogenic differentiation, while the conditioned medium of myogenic cells isolated from aged donors failed to improve FAPs proliferation and prevent adipogenic differentiation [80].

Ageing is also associated with comorbidities affecting FAPs behaviour. Particularly, there is a strong increase in the incidence of type 2 diabetes during ageing [137]. It has been shown that muscle regeneration is impaired in different mouse models of diabetes [138]. The ectopic fat deposition observed in regenerating muscle of diabetic mice was shown to originate from FAPs [138]. Insulin resistance in type 2 diabetes leads to the over-secretion of this hypoglycaemic hormone, which is a known inducer of adipogenic differentiation of FAPs *in vitro* [35].

Overall, intrinsic and extrinsic changes associated with ageing affect the regulation of FAPs, which, in turn, promote the accumulation of fibrofatty tissue and impair muscle regeneration. Further studies are required to evaluate the potential of therapeutic compounds targeting FAPs to rejuvenate aged skeletal muscles.

## 10. Conclusion

Skeletal muscle has a remarkable regenerative capacity that has been attributed to the presence and activity of muscle stem cells. The articles reviewed above clearly indicate that another type of stem cell, the FAPs, also plays a fundamental role in the regulation of skeletal muscle regeneration. This centrepiece position is attributable to their cellular crosstalks and secretion of paracrine factors that orchestrate inflammation and muscle stem cell functioning. The key to their success relies on their rapid and transient accumulation that is closely controlled by complex intrinsic and extrinsic factors. Excessive accumulation of FAPs and perturbations in their cell fate decision lead to fibrofatty deposition and impaired muscle regeneration in muscular disorders. Recent breakthroughs in single-cell transcriptomics allowed the identification of cellular heterogeneity in FAPs and their complex molecular interactome in the diverse stages of muscle regeneration and muscular disorders. These novel insights will play a key role in the development of new therapeutic avenues targeting FAPs to limit their accumulation and/or re-establish their function in order to reduce fibrofatty deposition and promote muscle regeneration in muscular disorders.

## Data accessibility

This article has no additional data.
